# The Impact of Exercise and Health Management on Workplace Creativity

**DOI:** 10.14789/jmj.JMJ23-0030-OA

**Published:** 2023-12-22

**Authors:** YASUYUKI HOCHI, MOTOKI MIZUNO

**Affiliations:** 1 Faculty of Health and Sports Science, Juntendo University, Chiba, Japan; 1 Faculty of Health and Sports Science, Juntendo University, Chiba, Japan; 2 Japan Women's College of Physical Education, Tokyo, Japan; 2 Japan Women's College of Physical Education, Tokyo, Japan; 3 Graduate School of Health and Sports Science, Juntendo University, Chiba, Japan; 3 Graduate School of Health and Sports Science, Juntendo University, Chiba, Japan

**Keywords:** employee health, workplace creativity, exercise, health and productivity management

## Abstract

**Objectives:**

This study aimed to investigate Japan’s service sector employees to determine whether employee creativity is associated with the provision of a fitness program (that encourages employees to perform physical exercise) or a health and productivity management (H&PM) program at the workplace.

**Design:**

This was a cross-sectional study.

**Methods:**

A nationwide online survey was conducted using stratified sampling. Data were obtained for respondents’ demographic characteristics, subjective health, exercise frequency, and organizational wellness support. Workplace creativity, psychological safety, and leadership were evaluated using standardized scales. A binary logistic regression was performed to examine the relationship between organizational wellness support programs and workplace creativity.

**Results:**

Respondents were 1,955 full-time employees in private-sector organizations (979 men and 976 women; mean age 40.30 ± 10.85). Workplace creativity was significantly more likely respondents whose employers provided a fitness program (*adjusted OR* = 1.86, *95% CI* = 1.39-2.48, *p*<.001) or an H&PM program (*adjusted OR* = 2.07, *95% CI* = 1.53-2.80, *p*<.001). Furthermore, workplace creativity was significantly more likely in employees who perceived themselves as rather healthy or healthy. Employees who exercised frequently over the past year were more likely to display creativity than those never exercised.

**Conclusions:**

Workplace creativity was associated with good subjective health, high exercise frequency, and organizational wellness support programs offered by employers. Human resource management for employees’ fitness and health is crucial for cultivating the productivity and innovation necessary for business success.

## Introduction

In Japan, the Third Sport Basic Plan was formulated on March 25, 2022. The new plan outlines the specific policies and targets to be advanced by the government and other organizations over the next five years (2022/4 to 2027/3)^1)^. The plan has been designed to ensure the legacy of the Tokyo 2020 Olympic and Paralympic Games and to facilitate the growth of the sports industry among others. However, for Japan to achieve economic growth, a major hurdle must be overcome: low productivity amid a lack of innovation^2)^. This is a particularly important issue in Japan's industry (the service sector)^2)^, which employs around 70% of the country's working population and encompasses the sports and health sectors^3)^. Moreover, as an impediment to innovation, around half of the companies in a government survey revealed that they lacked talented workers^2)^. While there is a growing focus on using digital technology in the workplace, Furukawa et al. notes that human knowledge and human skills are all the more important in a volatile business environment^4)^. Amabile et al. defined creativity as the production of novel and useful ideas in any domain, and innovation as the successful implementation of creative ideas within an organization^5)^. In this light, individual and team creativity is a starting point for innovation. Considerable evidence suggests that employee creativity can substantially contribute to organizational innovation, effectiveness, and survival^5-7)^. When employees exhibit creativity at work, they produce novel, and potentially useful, ideas about organizational products, practices, services, or procedures^5, 8)^. Thus, in the workplace, both individual and team creativity is essential for addressing all manner of workplace problems; as such, it will become an increasingly important priority for all organizational layers in the service sector^9)^. Therefore, there is an increasing need for a greater understanding of the contextual factors that may enhance or diminish employees' creativity as well as the interaction between personal characteristics and work environment^8-10)^. Further, it is important to identify the role of leadership in encouraging employee creativity^9, 10)^. Another concept gaining increasing attention in the corporate sector is psychological safety (as defined by Edmondson, 1999)^11)^. Studies have shown that psychological safety correlates positively with creativity, and that effective leadership facilitates both psychological safety and employee creativity in the workplace^12-16)^.

Japan has shown interest in organizational support for employee wellness, inspired by the ideas in The Healthy Company (Rosen, 1992)^17)^. In this context, Japan sports agency（MEXT） led a wellness program named the Sports Yell Company program^18)^. Alongside this, there are company-level “health and productivity management” (H&PM) programs, which align with a model of employee wellness promoted by the Ministry of Economy, Trade and Industry (METI)^19)^. Previous studies have revealed that, companies with an effective H&PM program tend to have better stock performance^20, 21)^. Given the well-demonstrated association between employee health (as measured by absenteeism, for instance) and productivity (as measured by presenteeism, for instance), greater corporate interest in employee health will lead to more positive outcomes. However, thus far, the literature has focused on outcomes such as return on (or loss on) investment in health, productivity measures, and health problems^22-31)^; few studies have measured creativity in a workplace context or changes in employee attitudes and behaviors. Some reports suggest that physical exercise positively affects creative thinking, creative idea generation, and cognitive processing^32, 33)^. However, systematic reviews concluded that further research is necessary to obtain clear evidence of the association between exercise and creativity^34, 35)^. The present study was motivated by the following idea: if it was demonstrated that employee creativity is greater in workplaces with wellness programs, this would offer the corporate sector a greater incentive to invest in human resource management for employee health, ultimately leading to greater innovation.

To this end, we conducted a cross-sectional study on employees in the service sector to determine whether employee creativity is associated with the provision of either one or both of the following types of organizational wellness support: a fitness program (a program encouraging employees to engage in physical exercise) and an H&PM program.

## Method

### Sample and procedure

A nationwide online survey was conducted using stratified sampling, including individuals who were aged 20-59 and employed full time in private-sector organizations, classified as tertiary industry (service sector) employers by the Ministry of Internal Affairs and Communications. The study sample comprised participants selected from the panel registered with Cross Marketing, Inc., a leading marketing research agency in Japan. The survey was conducted between October and November 2022. Before starting the online survey, the participants were presented with a webpage detailing the rationale, objective of the survey, ethical considerations and stating that their anonymity would be safeguarded. The participants were then invited to click on an “agree” button to indicate their informed consent to participate in the survey.

Survey data cleaning was performed to eliminate data from respondents whose responses could bias the results. Specifically, we identified and removed straight liners (respondents who chose the same answer option repeatedly) and set a trick question (a question designed to “trap” respondents who were not paying attention).

### Measures

#### Participant characteristics

The following participant attributes were obtained: gender, age, education (highest level of education), marital status, parental status (whether the person has any children), age of the youngest (or only) child (if any), managerial status (whether the person has a management title), work hours, occupation, subjective health, exercise frequency, and whether wellness support was provided in the workplace. Subjective health was rated on a 4-point scale, with the following answer options: unhealthy, rather unhealthy, rather healthy, and healthy. For exercise frequency, respondents described how frequently they had engaged in an exercise session lasting at least 30 minutes, over the past year; the answer options were: never, one to three days a year, four to eleven days a year, one to three days a month, one to two days a week, and at least three days a week. To determine whether organizational wellness support was provided, the following questions were presented: “Does your workplace provide a fitness program?” and “Does your workplace provide a H&PM program?” In both cases, respondents could answer “yes,” “no,” or “unsure.”

#### Workplace creativity

Creativity in the workplace was measured using a six-item scale by Maruyama and Fuji (2022)^36)^, based on the original scale by Carmeli and Schaubroeck (2007) that measures creative involvement at work^10)^. Each item contained a statement rated on a 7-point scale to indicate level of agreement (absolutely disagree to strongly agree). The items (as translated from Japanese) were as follows: “I exhibit originality at work,” “I take risks to generate new ideas needed in my work,” “I'm prepared to change existing work procedures and methods and I look for new ways to use equipment,” “I resolve the issues that entail difficulties,” “I try out new ideas when approaching problems,” and “I generate ideas that are unprecedented but useful for work.” Based on their total scores for the six items, the respondents were divided into quartiles, with the top 25% categorized as the high creativity group and the remaining 75% as the non-high creativity group.

#### Workplace culture and leadership

The previous study suggests that employees' creativity is moderated by contextual factors such as workplace culture and leadership, as well as by intra-individual factors^8, 9)^. To evaluate workplace culture, we used Edmondson's (1999) Team Psychological Safety Scale^11)^. To assess leadership, we used Walumbwa et al.'s (2008) Authentic Leadership Questionnaire^37)^. The Team Psychological Safety Scale consists of seven items, each rated on a 7-point scale (from “completely disagree” to “absolutely agree”). The items were prefaced with the following statement: “We will ask you about your team (workplace, department, store members) with which you usually work.” The Authentic Leadership Questionnaire consists of 16 items, each rated on a 5-point scale (from “not at all” to “frequently, if not always”) and prefaced with the following statement: “The following survey items refer to your leader's style, as you perceive it. Judge how frequently each statement fits his or her leadership style using the following scale.”

### Ethical considerations

This study was approved by the research ethics committee of the Japan Women's College of Physical Education (Application Number: 2022-20).

### Statistical analysis

A binomial logistic regression was performed, with the explanatory variables being subjective health, exercise frequency, and organizational wellness support, and the objective variable being workplace creativity. We also set several moderator variables (confounders) on the premise that the variables in question could affect workplace creativity directly or indirectly. These moderator variables included: gender, age, education, marital status, parental status, managerial status, work hours, occupation, psychological safety, and authentic leadership. Using the adjusted odds ratios for these items, we could control the confounding factors. IBM SPSS version 28 for Windows was used for statistical analysis.

## Results

### Participant attributes

Table 1 shows the attributes of participants. The cleaned survey data comprised 1,955 respondents employed full-time in private-sector organizations classified as tertiary industry companies. In total, there were 979 men (50.1%) and 976 women (49.9%); 958 (48.0%) respondents were married. Furthermore, 673 (34.4%) respondents had children; the youngest (or only) child was aged below 6 in 206 (10.5%) cases, 6-11 in 118 (6.0%) cases, 12-17 in 140 (7.2%) cases, and 18 or above in 209 (10.7%) cases. The respondents' mean age was 40.30 ± 10.85; 485 (24.8%) were in their 20s, 486 (24.9%) in their 30s, 488 (25.0%) in their 40s, and 496 (25.4%) in their 50s. Regarding education level, 384 (19.6%) had completed high school, 226 (11.6%) technical/vocational college, 169 (8.6%) junior college (or vocational high school), 1,077 (55.1%) university (four-year undergraduate program), 66 (3.4%) a master's program, 11 (0.6%) a doctoral program, and 22 (1.1%) other. Regarding managerial status, 598 (30.6%) participants had a management title, while 1,357 (69.4%) were general employees with no management title. Regarding work hours per week, 1,349 (69.0%) participants worked for less than 45 hours, 462 (23.6%) worked between 45 and 54 hours, 103 (5.3%) worked between 55 and 64 hours, and 41 (2.1%) worked for 65 hours or longer. Regarding occupation, 301 (15.4%) participants worked in sales, 549 (28.1%) in admin/accounts, 44 (2.3%) in HR, 57 (2.9%) in planning/marketing, 60 (3.1%) in senior or middle management, 269 (13.8%) in customer services, 286 (14.6%) in specialized services, 184 (9.4%) in technical services, and 205 (10.5%) in others.

**Table 1:** Differences in workplace creativity according to the participants' attributes

		Non-high Creativity (n=1,453)			High Creativity (n=502)		
		n	%		n	%	χ^2^
Gender	Male	688	47.4%		291	58.0%	16.83**
	Female	765	52.6%		211	42.0%	
Marital status	Unmarried	769	52.9%		228	45.4%	8.41*
	Married	684	47.1%		274	54.6%	
Parental status	No children	978	67.3%		304	60.6%	15.65*
	< 6 years old	156	10.7%		50	10.0%	
	6-11 years old	84	5.8%		34	6.8%	
	12-17 years old	87	6.0%		53	10.6%	
	≥ 18 years old	148	10.2%		61	12.2%	
Age	20s	383	26.4%		102	20.3%	8.90*
	30s	362	24.9%		124	24.7%	
	40s	356	24.5%		132	26.3%	
	50s	352	24.2%		144	28.7%	
Education	High school	307	21.1%		77	15.3%	40.36**
	Technical/vocational college	176	12.1%		50	10.0%	
	Junior college (or vocational high school)	140	9.6%		29	5.8%	
	University (four-year undergraduate program)	765	52.6%		312	62.2%	
	Master's program	40	2.8%		26	5.2%	
	Doctoral program	4	0.3%		7	1.4%	
	Other	21	1.4%		1	0.2%	
Managerial status	Yes	378	26.0%		220	43.8%	55.74**
	No (general employee)	1,075	74.0%		282	56.2%	
Working hours (per week)	< 45 h	1,023	70.4%		326	64.9%	9.27*
	45 h-54 h	337	23.2%		125	24.9%	
	55 h-64 h	67	4.6%		36	7.2%	
	≥ 65 h	26	1.8%		15	3.0%	
Occupation	Sales	212	14.6%		89	17.7%	62.14**
	Admin/accounts	445	30.6%		104	20.7%	
	Human resources	26	1.8%		18	3.6%	
	Planning/marketing	29	2.0%		28	5.6%	
	Senior/middle management	30	2.1%		30	6.0%	
	Customer services	211	14.5%		58	11.6%	
	Specialized services	202	13.9%		84	16.7%	
	Technical services	137	9.4%		47	9.4%	
	Other	161	11.1%		44	8.8%	

**p<.01, *p<.05.

### Exercise frequency and organizational wellness support

Table 2 shows the exercise frequency and organizational wellness support of participants. Regarding subjective health, 140 (7.2%) respondents answered “unhealthy,” 330 (16.9%) “rather unhealthy,” 966 (49.4%) “rather healthy,” and 519 (26.5%) “healthy.” When asked how frequently they had engaged in an exercise session lasting at least 30 minutes over the past year, 879 (45.0%) answered never, 175 (9.0%) answered one to three days a year, 66 (3.4%) answered four to 11 days a year, 154 (7.9%) answered one to three days a month, 415 (21.2%) answered one to two days a week, and 266 (13.6%) answered at least three days a week. When asked whether their workplace provided a fitness program, 1,393 (71.3%) participants said no, 294 (15.0%) said yes, and 268 (13.7%) were unsure. When asked whether their workplace provided a H&PM program, 1,030 (52.7%) participants answered no, 280 (14.3%) answered yes, and 645 (33.0%) were unsure.

**Table 2:** Differences in workplace creativity according to exercise frequency and health management

		Non-high Creativity (n=1,453)			High Creativity (n=502)		
		n	%		n	%	χ^2^
Subjective health	Unhealthy	122	8.4%		18	3.6%	18.92**
	Rather unhealthy	256	17.6%		74	14.7%	
	Rather healthy	711	48.9%		255	50.8%	
	Healthy	364	25.1%		155	30.9%	
Exercise frequency	Never	717	49.3%		162	32.3%	50.39**
	1-3 days/year	131	9.0%		44	8.8%	
	4-11 days/year	45	3.1%		21	4.2%	
	1-3 days/month	103	7.1%		51	10.2%	
	1-2 days/week	286	19.7%		129	25.7%	
	≥ 3 days/week	171	11.8%		95	18.9%	
Promote fitness activities	No	1,082	74.5%		311	62.0%	60.14**
	Yes	165	11.4%		129	25.7%	
	Unsure	206	14.2%		62	12.4%	
Health and productivity management program	No	813	56.0%		217	43.2%	70.97**
	Yes	152	10.5%		128	25.5%	
	Unsure	488	33.6%		157	31.3%	
Psychological safety	M (±SD）	4.52	（±.689）		5.03	（±.790）	**
Authentic leadership	M (±SD）	2.90	（±.754）		3.26	（±.849）	**

^a^**p<.01, *p<.05. ^b^ The psychological safety and the Authentic Leadership were using Welch’s t-test.

### Creativity at the workplace

A total of 502 respondents were classed as creative, their total scores falling in the top quartile of scores (*M* = 30.48 ± 3.27), while 1,453 were classed as uncreative (*M* = 17.53 ± 6.39). The two groups were compared regarding their basic attributes using a chi-squared test. The analysis revealed significant inter-group differences for gender, age, education, marital status, parental status, whether the respondent had a managerial status, work hours, and occupation (Table 1).

The two groups also differed significantly on all items of subjective health, exercise frequency, whether a fitness activity was promoted, whether an H&PM program was promoted, Team Psychological Safety Scores, and Authentic Leadership Questionnaire scores (Table 2). For the team psychological safety scale and the Authentic Leadership Questionnaire, following the precedent set in the literature, using Welch's t-test.

### Factors related to employee creativity in the workplace.

Table 3 shows the results of the binomial logistic regression analysis. Employee creativity in the workplace was significantly more likely in respondents who perceived themselves as rather healthy (*adjusted OR* = 2.14, *95% CI* = 1.23-3.71, *p*<.01) or healthy (*adjusted OR* = 2.09, *95% CI* = 1.18-3.70, *p*<.05) than those who perceived themselves as unhealthy. Furthermore, workplace creativity was significantly more likely in respondents who engaged in an exercise session lasting at least 30 minutes for one to three days a month (*adjusted OR* = 1.90, *95% CI* = 1.26-2.86, *p*<.01), one to two days a week (*adjusted OR* = 1.69, *95% CI* = 1.26-2.27, *p*<.001), or at least three days a week (*adjusted OR* = 1.76, *95% CI* = 1.27-2.46, *p*<.001), than by those who had never exercised in the past year. Of the exercise frequency categories, “at least three days a week” was associated with the greatest increase in adjusted odds ratio. In addition, workplace creativity was significantly more likely in respondents whose employers promoted fitness activities (*adjusted OR* = 1.86, *95% CI* = 1.39-2.48, *p*<.001) and those whose employers provided an H&PM program (*adjusted OR* = 2.07, *95% CI* = 1.53-2.80, *p*<.001) than in respondents whose employers did not provide either.

**Table 3:** Results of the binomial logistic regression analysis

		Crude OR	(*95%CI*)	*P*-value	Adjusted *OR*	(*95%CI*)	*P*-value
Subjective health	Unhealthy	1.00	(*Reference*)		1.00	(*Reference*)	
	Rather unhealthy	1.96	(*1.12-3.42*)	*	1.80	(*0.99-3.27*)	*n.s.*
	Rather healthy	2.43	(*1.45-4.07*)	**	2.14	(*1.23-3.71*)	**
	Healthy	2.89	(*1.70-4.90*)	**	2.09	(*1.18-3.70*)	*
Exercise frequency	Never	1.00	(*Reference*)		1.00	(*Reference*)	
	1-3 days/year	1.49	(*1.01-2.18*)	*	1.39	(*0.93-2.09*)	*n.s.*
	4-11 days/year	2.07	(*1.20-3.56*)	**	1.54	(*0.86-2.75*)	*n.s.*
	1-3 days/month	2.19	(*1.50-3.19*)	**	1.90	(*1.26-2.86*)	**
	1-2 days/week	2.00	(*1.53-2.61*)	**	1.69	(*1.26-2.27*)	**
	≥ 3 days/week	2.46	(*1.82-3.33*)	**	1.76	(*1.27-2.46*)	**
Promote fitness activities	No	1.00	(*Reference*)		1.00	(*Reference*)	
	Yes	0.96	(*0.70-1.30*)	*n.s.*	1.86	(*1.39-2.48*)	**
	Unsure	2.60	(*1.80-3.74*)	**	1.03	(*0.74-1.43*)	*n.s.*
Health and productivity management program	No	1.00	(*Reference*)		1.00	(*Reference*)	
	Yes	3.15	(*2.39-4.17*)	**	2.07	(*1.53-2.80*)	**
	Unsure	1.21	(*0.95-1.52*)	*n.s.*	1.03	(*0.80-1.33*)	*n.s.*

^a^ **p<.01, *p<.05; *n.s.*=non-significant; *OR* = Odds ratio; *95% CI* = 95% confidence interval. ^b^ Objective variable: workplace creativity (0 = non-high creativity, 1 = high creativity). ^c^ The adjusted OR were intended to control for the following: gender, marital status, parental status, age, education, managerial status, work hours, occupation, psychological safety, and authentic leadership.

### Discussion

This study examined survey data from employees in the service sector to reveal how employee creativity is associated with exercise and organizational wellness support. Having controlled for certain respondent attributes that could affect employee creativity (gender, age, education, marital status, parental status, managerial status, work hours, occupation, psychological safety, and authentic leadership), we found that employees' creativity in the workplace is associated with their subjective health and whether the employer provides a fitness program or an H&PM program. To the best of our knowledge, this finding constitutes the first scientific evidence of such an association.

### Exercise and workplace creativity

Our survey revealed that, over the past year, 45% (879) of the respondents had never engaged in an exercise session lasting at least 30 minutes, while 34.7% (681) had done so at least one day a week. In a 2022 survey on engagement in sport, the Japan Sports Agency found that 47% of Japanese men and women aged 20-59 engage in exercise at least one day a week. However, the agency's exercise data pertained to general members of the public, who may or may not have been employed and who could have been employed in any kind of job; the value of our data lies in the fact that it specifically highlights exercise frequency among people employed full-time in Japan's private sector. Our analysis revealed that workplace creativity was significantly more likely to be exhibited by employees who perceived themselves as rather than by those who perceived themselves as unhealthy. The literature on the relationship between employee health and workplace productivity has shown that effective organizational support for employee wellness, as measured by a decline in presenteeism among other things, leads to higher workplace creativity. Creativity was also significantly more likely to be exhibited by employees engaged in an exercise session lasting at least 30 minutes on one to three days a month (*adjusted OR* = 1.90, *95% CI* = 1.26-2.86, *p*<.01), one to two days a week (*adjusted OR* = 1.69, *95% CI* = 1.26-2.27, *p*<.001), or at least three days a week (*adjusted OR* = 1.76, *95% CI* = 1.27-2.46, *p*<.001) over the past year, than by those who had never exercised in the past year; furthermore, the highest adjusted odds ratio was found for those exercised “at least three days a month. The Government of Japan is committed to the goal of having 70% of the adult population engage in exercise at least one day a week and having 50% engage in exercise at least three days a week. In this regard, our findings offer important insights for employees in the service sector; exercising one to three days a month is more effective for facilitating creativity in the workplace than not exercising at all. Other studies suggest that exercise can facilitate workplace creativity even at a low frequency, provided that the physical activity is regular. Specifically, one report suggested that even a single exercise session can facilitate creative thinking^32)^; other studies showed that habitual physical activity has a moderate effect on creative ideation, and that chronic physical activity interventions enhance creativity^33-35)^.

### Organizational wellness support and creativity

Our second main finding concerns the role of organizational wellness support in employees' creativity. We found that workplace creativity was significantly more likely to occur among respondents whose employers promoted fitness activity (*adjusted OR* = 1.86, *95% CI* = 1.39-2.48, *p*<.001) than among those whose employers did not. Furthermore, creativity was significantly more likely to occur among respondents whose employers provided an H&PM program (*adjusted OR* = 2.07, *95% CI* = 1.53-2.80, *p*<.001) than among those whose employers did not. Admittedly, these results offer no precise insights into how such programs contribute to individual employees' behaviors and health condition. Nonetheless, they do corroborate the claim made in “The Healthy Company” that companies with healthier workforces are more profitable; that is, individual and team creativity constitutes both the starting point for innovation and a process for resolving problems. There is extensive evidence showing that employee creativity facilitates organizational innovation and organizational effectiveness^6, 7)^.

Finally, we discuss the results in Table 2. Japan has shown increasing interest in having the corporate sector execute integrated strategies for encouraging employees to engage in physical and health- supporting activities, as evidenced by government- led programs such as the Sports Yell Company program^18)^ and the H&PM program^19)^. However, our results suggest that the government's employee wellness agenda is yet to gain traction; in our survey, few companies provided a fitness or H&PM program for employees. As stated in the Introduction section, a report suggested that the greatest impediment to innovation is a lack of talented workers within the company^2)^. In today's volatile, uncertain, complex, and ambiguous business environment, it is all the more essential to provide a workplace and talent management strategy that unleashes employees' creativity. While it is true that profit growth will always be paramount to business survival, and that employers are sometimes unable to invest enough in talent development and employee welfare, sacrificing employee wellness for the sake of immediate profit is the wrong approach. Although investing in employee wellness yields no instant or immediately apparent return on investment, organizational support for employees' fitness and health plays a crucial role in cultivating the productivity and innovation necessary for business success in a volatile, uncertain, complex, and ambiguous environment.

### Conclusions

Using survey data pertaining to 1,955 full-time employees in Japan's service sector, we conducted a cross-sectional study to examine the relationship between organizational wellness support at the workplace, exercise frequency, and workplace creativity. Based on the results, three conclusions can be drawn. First, creativity in the workplace is associated with subjective health: compared with those who perceived themselves as unhealthy, respondents who perceived themselves as healthy had increased adjusted odds ratios from 2.09 to 2.14 for demonstrating workplace creativity. Second, workplace creativity is associated with habitual exercise: compared with those who had never exercised in the past year, respondents who performed exercise showed increased workplace creativity, with adjusted odds ratios of 1.90 for those who exercised for one to three days a month, 1.69 for those who exercised for one to two days a week, and 1.76 for those who exercised at least three days a week. Third, workplace creativity is associated with organizational wellness support: provision of a fitness program and an H&PM program by employers was associated with increased adjusted odds ratios of 1.86 and 2.07, respectively, for workplace creativity. Thus, human resource management for employees' fitness and health is crucial for cultivating the productivity and innovation necessary for business success.

### Limitations and future research

This study adopted a cross-sectional approach and analyzed survey data pertaining to a single time-point. As such, the findings warrant no conclusions about any direct causal relationship that exercise and organizational wellness support may have with workplace creativity (the interaction mechanism). Furthermore, we believe that the impact of company size, such as number of employees and capitalization, should also be taken into consideration. When interpreting the results, the following precautions should be observed. First, as respondents rated their own creativity, we cannot rule out the possibility that their self-reports were affected by cognitive bias (such as the Dunning-Kruger effect). Thus, workplace creativity as measured in our study may not accurately reflect actual behaviors and outcomes in everyday workplace activities. Second, as respondents were asked about their exercise frequency over the past year, there is a possibility that their responses were influenced by recall bias, too. Many complex and latent factors moderate employee behavior in the workplace. By combining survey data with objective data from wearable devices or physiological measures, it is possible to deliver more reliable evidence for the effects of exercise and organizational wellness support on employees. Rigorously conducted randomized controlled intervention studies and more cross-sectional studies are needed to broaden the evidence in this nascent field of research.

## Funding

No funding was received.

## Author contributions

YH and MM conceived the idea of the study. YH developed the statistical analysis plan and conducted statistical analyses. YH and MM contributed to the interpretation of the results. YH drafted the original manuscript. MM supervised the conduct of this study. All authors reviewed the manuscript draft and revised it critically on intellectual content. All authors approved the final version of the manuscript to be published.

## Conflicts of interest statement

The authors declare that they have no conflicts of interest.

## References

[1] Ministry of Education, Culture, Sports, Science and Technology; Japan's Third Sport Basic Plan. https://www.mext.go.jp/sports/en/index.htm (Last accessed at September 1, 2023)

[2] National Institute of Science and Technology Policy: Report on the Japanese National Innovation Survey 2020 (J-NIS 2020). NISTEP REPORT, No.192. https://doi.org/10.15108/nr192 (Last accessed at September 1, 2023) (in Japanese)

[3] Statistics Bureau of Japan, Annual Report on the Labour Force Survey 2022. https://www.stat.go.jp/english/data/roudou/report/2022/index.html (Last accessed at September 1, 2023)

[4] Furukawa Y, Sato Y: The fundamental survey of the productivity of white-collar workers. Mita business review, 2005; 49: 97-119. (in Japanese)

[5] Amabile TM, Conti R, Coon H, Lazenby J, Herron M: Assessing the work environment for creativity. Academy of management journal, 1996; 39: 1154-1184.

[6] Amabile TM, Pratt MG: The dynamic componential model of creativity and innovation in organizations: Making progress, making meaning. Research in organizational behavior, 2016; 36: 157-183.

[7] Nonaka I: The knowledge-creating company. Harvard Business Review, 1991; 69: 96-104.

[8] Shalley CE, Zhou J, Oldham GR: The effects of personal and contextual characteristics on creativity: Where should we go from here? Journal of management, 2004; 30: 933-958.

[9] Shalley CE, Gilson LL: What leaders need to know: A review of social and contextual factors that can foster or hinder creativity. Leadership Quarterly, 2004; 15: 33-53.

[10] Carmeli A, Schaubroeck J: The influence of leaders' and other referents’ normative expectations on individual involvement in creative work. The Leadership Quarterly, 2007; 18: 35-48.

[11] Edmondson A: Psychological safety and learning behavior in work teams. Administrative science quarterly, 1999; 44: 350-383.

[12] Frazier ML, Fainshmidt S, Klinger RL, Pezeshkan A, Vracheva V: Psychological safety: A meta‐analytic review and extension. Personnel psychology, 2017; 70: 113-165.

[13] Carmeli A, Reiter-Palmon R, Ziv E: Inclusive leadership and employee involvement in creative tasks in the workplace: The mediating role of psychological safety. Creativity Research Journal, 2010; 22: 250-260.

[14] Müceldili B, Turan H, Erdil O: The influence of authentic leadership on creativity and innovativeness. Procedia-Social and Behavioral Sciences, 2013; 99: 673-681.

[15] Mumford MD, Scott GM, Gaddis B, Strange JM: Leading creative people: Orchestrating expertise and relationships. The leadership quarterly, 2002; 13: 705-750.

[16] Jung DI, Chow C, Wu A: The role of transformational leadership in enhancing organizational innovation: Hypotheses and some preliminary findings. The leadership quarterly, 2003; 14: 525-544.

[17] Rosen R, Berger L: The Healthy Company: Eight Strategies to Develop People, Productivity, and Profits. New York: TarcherPerigee, 1992.

[18] Ministry of Education, Culture, Sports, Science and Technology; Sports Yell Company. https://www.mext.go.jp/sports/en/b_menu/policy/sportsnation/yellcompany.htm (Last accessed at September 1, 2023)

[19] Ministry of International Trade and Industry; Health and Productivity management. https://www.meti.go.jp/policy/mono_info_service/healthcare/kenko_keiei.html (Last accessed at September 1, 2023)

[20] Goetzel RZ, Fabius R, Fabius D, et al: The Stock Performance of C. Everett Koop Award Winners Compared With the Standard & Poor's 500 Index. J Occup Environ Med, 2016; 58: 9-15. 26716843 10.1097/JOM.0000000000000632 PMC4697959

[21] Fabius R, Loeppke RR, Hohn T, et al: Tracking the Market Performance of Companies That Integrate a Culture of Health and Safety: An Assessment of Corporate Health Achievement Award Applicants. J Occup Environ Med, 2016; 58: 3-8. 26716842 10.1097/JOM.0000000000000638

[22] Parks KM, Steelman LA: Organizational wellness programs: a meta-analysis. J Occup Health Psychol, 2008; 13: 58-68. 18211169 10.1037/1076-8998.13.1.58

[23] Pauly MV, Nicholson S, Xu J, et al: A general model of the impact of absenteeism on employers and employees. Health Econ, 2002; 11: 221-231. 11921319 10.1002/hec.648

[24] Nicholson S, Pauly MV, Polsky D, Sharda C, Szrek H, Berger ML: Measuring the effects of work loss on productivity with team production. Health Econ, 2006; 15: 111-123. 16200550 10.1002/hec.1052

[25] Goetzel RZ, Long SR, Ozminkowski RJ, Hawkins K, Wang S, Lynch W: Health, absence, disability, and presenteeism cost estimates of certain physical and mental health conditions affecting U.S. employers. J Occup Environ Med, 2004; 46: 398-412. 10.1097/01.jom.0000121151.40413.bd 15076658

[26] Loeppke R, Taitel M, Haufle V, Parry T, Kessler RC, Jinnett K: Health and productivity as a business strategy: a multiemployer study. J Occup Environ Med, 2009; 51: 411-428. 19339899 10.1097/JOM.0b013e3181a39180

[27] Mitchell RJ, Bates P: Measuring health-related productivity loss. Popul Health Manag, 2011; 14: 93-98. 21091370 10.1089/pop.2010.0014 PMC3128441

[28] Wada K, Arakida M, Watanabe R, Negishi M, Sato J, Tsutsumi A: The economic impact of loss of performance due to absenteeism and presenteeism caused by depressive symptoms and comorbid health conditions among Japanese workers. Ind Health, 2013; 51: 482-489. 23892900 10.2486/indhealth.2013-0016 PMC4202740

[29] Suzuki T, Miyaki K, Song Y, et al: Relationship between sickness presenteeism (WHO-HPQ) with depression and sickness absence due to mental disease in a cohort of Japanese workers. J Affect Disord, 2015; 180: 14-20. 25879720 10.1016/j.jad.2015.03.034

[30] Nagata T, Mori K, Ohtani M, et al: Total Health-Related Costs Due to Absenteeism, Presenteeism, and Medical and Pharmaceutical Expenses in Japanese Employers. J Occup Environ Med, 2018; 60: e273-e280. 29394196 10.1097/JOM.0000000000001291 PMC5959215

[31] Zhang W, Sun H, Woodcock S, Anis A: Illness related wage and productivity losses: Valuing ‘presenteeism’. Soc Sci Med, 2015; 147: 62-71. 26547046 10.1016/j.socscimed.2015.10.056

[32] Steinberg H, Sykes EA, Moss T, Lowery S, LeBoutillier N, Dewey A: Exercise enhances creativity independently of mood. Br J Sports Med, 1997; 31: 240-245. 9298561 10.1136/bjsm.31.3.240 PMC1332529

[33] Bollimbala A, James PS, Ganguli S: The impact of physical activity intervention on creativity: Role of flexibility vs persistence pathways. Thinking Skills and Creativity, 2023; 49: 101313.

[34] Frith E, Ryu S, Kang M, Loprinzi PD: Systematic Review of the Proposed Associations between Physical Exercise and Creative Thinking. Eur J Psychol, 2019; 15: 858-877. 33680164 10.5964/ejop.v15i4.1773 PMC7909196

[35] Rominger C, Schneider M, Fink A, Tran US, Perchtold-Stefan CM, Schwerdtfeger AR: Acute and Chronic Physical Activity Increases Creative Ideation Performance: A Systematic Review and Multilevel Meta-analysis. Sports Med Open, 2022; 8: 62. 35523914 10.1186/s40798-022-00444-9 PMC9076802

[36] Maruyamura J, Fuji K: Effects of workplace humor on the creativity at work: Mediating role of psychological safety. Japanese Association of Industrial/Organizational Psychology Journal, 2022; 35: 381-392. (in Japanese)

[37] Walumbwa FO, Avolio BJ, Gardner WL, Wernsing TS, Peterson SJ: Authentic leadership: Development and validation of a theory-based measure. Journal of management, 2008; 34: 89-126.

